# Supplementary material for: Picoplankton nitrogen guilds in the tropical and subtropical oceans: From the surface to the deep
Source: PLoS One. 2025 Nov 5;20(11):e0335222. doi: 10.1371/journal.pone.0335222 (PMC12588538; doi:10.1371/journal.pone.0335222)
Supplement: S1 File — (ZIP) [file pone.0335222.s001.zip › supplementary info/N_cycle/G_ilvC/Lemaire_2021_biomolecules-11-01679.pdf]

biomolecules

Communication

Structural Rearrangements of a Dodecameric Ketol-Acid Reductoisomerase Isolated from a Marine Thermophilic Methanogen

Olivier Nicolas Lemaire [1], Marie-Caroline Müller [1], Jörg Kahnt [2] and Tristan Wagner [1,*]

[1] Microbial Metabolism Research Group, Max Planck Institute for Marine Microbiology, Celsiusstraße 1, 28359 Bremen, Germany; olemaire@mpi-bremen.de (O.N.L.); mmueller@mpi-bremen.de (M.-C.M.)
[2] Core Facility for Mass Spectrometry & Proteomics, Max Planck Institute for Terrestrial Microbiology, Karl-von-Frisch-Straße 10, 35043 Marburg, Germany; kahnt@mpi-marburg.mpg.de
* Correspondence: twagner@mpi-bremen.de

Abstract: Ketol-acid reductoisomerase (KARI) orchestrates the biosynthesis of branched-chain amino acids, an elementary reaction in prototrophic organisms as well as a valuable process in biotechnology. Bacterial KARIs belonging to class I organise as dimers or dodecamers and were intensively studied to understand their remarkable specificity towards NADH or NADPH, but also to develop antibiotics. Here, we present the first structural study on a KARI natively isolated from a methanogenic archaea. The dodecameric structure of 0.44-MDa was obtained in two different conformations, an open and close state refined to a resolution of 2.2-Å and 2.1-Å, respectively. These structures illustrate the conformational movement required for substrate and coenzyme binding. While the close state presents the complete NADP bound in front of a partially occupied Mg^{2+}-site, the Mg^{2+}-free open state contains a tartrate at the nicotinamide location and a bound NADP with the adenine-nicotinamide protruding out of the active site. Structural comparisons show a very high conservation of the active site environment and detailed analyses point towards few specific residues required for the dodecamerisation. These residues are not conserved in other dodecameric KARIs that stabilise their trimeric interface differently, suggesting that dodecamerisation, the cellular role of which is still unknown, might have occurred several times in the evolution of KARIs.

Keywords: ketol-acid reductoisomerases; methanogenic archaea; X-ray crystallography; conformational rearrangement; native purification; oligomerisation

check for updates

Citation: Lemaire, O.N.; Müller, M.-C.; Kahnt, J.; Wagner, T. Structural Rearrangements of a Dodecameric Ketol-Acid Reductoisomerase Isolated from a Marine Thermophilic Methanogen. *Biomolecules* **2021**, *11*, 1679. https://doi.org/10.3390/biom11111679

Academic Editor: Victoria Bunik

Received: 14 October 2021
Accepted: 8 November 2021
Published: 11 November 2021

Publisher's Note: MDPI stays neutral with regard to jurisdictional claims in published maps and institutional affiliations.

Copyright: © 2021 by the authors. Licensee MDPI, Basel, Switzerland. This article is an open access article distributed under the terms and conditions of the Creative Commons Attribution (CC BY) license (https://creativecommons.org/licenses/by/4.0/).

1. Introduction

The ketol-acid reductoisomerase (KARI, EC 1.1.1.86) catalyses the reversible conversion of acetohydroxy acids into the corresponding hydroxyl valerates, a crucial step in the biosynthesis pathway of valine, leucine and isoleucine [1]. The enzyme is therefore necessary for amino acid prototrophy. Because of its importance in bacteria, plants and fungi, but its absence in metazoans, the enzyme receives increasing attention for the design of antibiotics, herbicides and fungicides [2–4]. On the other hand, branched-chain amino acids are valuable compounds for the food, cosmetic and pharmaceutical industries, with a constantly increasing global market (e.g., L-valine: average annual increase rate >5% [5,6]). Microbial fermentation is the first source of branched-chain amino acids worldwide. Hence, the study of the enzymes involved in their biosynthetic pathway is a far-reaching process, which explains the numerous characterised KARIs among prokaryotes.

KARIs are structurally composed of an N-terminal Rossmann domain and a C-terminal "knot" domain [1,7]. They can be distinguished in two classes: the short class I gathering dimeric and dodecameric enzymes, and the monomeric or tetrameric longer class II KARIs [1,6–14]. Prokaryotes can harbour class I or II enzymes, while eukaryotes only contain class II. Class I KARI was suggested to be the ancestral group from which the class

II arose by duplication of the knot domain [7]. Most KARIs are dependent on NADPH, which was proposed to be the ancestral trait [1,6,15,16]. However, NADH-dependent or bispecific enzymes were also discovered [8,13,15–17]. The use of NADH-dependent enzymes is preferred for biotechnological processes since the cellular content of NADH is higher than NADPH, a ratio that would stimulate the amino acid production rate. Therefore, attempts to engineer NADPH-dependent KARIs into NADH-dependent ones were performed, which brought a considerable amount of information regarding the residues involved in NAD(P)H selectivity [15–19].

Numerous structures of class I KARIs were obtained with Mg^{2+} ions, NAD(P)H, substrate analogues and/or inhibitors [6–8,10–13,16,17,20]. This deep and collective work highlighted some structural rearrangements triggered by binding of Mg^{2+} and NAD(P)H [12,13,17,20]. Binding of NAD(P)H was, however, established to provoke an induced fit mechanism triggering the conformational change from an open to a closed conformation. This closed conformation is catalytically competent and can bind the substrate on the two Mg^{2+} ions carried by the knot domain.

The hydrogenotrophic methanogen *Methanothermococcus thermolithotrophicus* (DSM 2095) is a marine thermophilic archaeon [21]. Its fast growth on minimal mineral medium gassed with H_2 and CO_2 makes this organism an excellent model to study chemolithoautotrophy, which implies the *de novo* biosynthesis of all amino acids. Therefore, KARI must have a predominant role in the branched-chain amino acid biosynthesis of this anaerobic archaeon.

Here we describe the structure of the purified native dodecameric enzyme from *M. thermolithotrophicus* (referred here as *Mt*KARI), which is the first structurally characterised KARI from a methanogen. The structural analyses of a close state containing Mg^{2+} and NADP, and an open state containing NADP and tartrate without Mg^{2+}, provide new insights into class I KARIs, as well as the evolution of the oligomeric states.

2. Materials and Methods

2.1. Cultivation of *M. thermolithotrophicus*

Methanothermococcus thermolithotrophicus strain DSM 2095 was obtained from the Deutsche Sammlung von Mikroorganismen und Zellkulturen (DSMZ, Braunschweig, Germany). The archaeon was cultivated in a fermenter at 65 °C and harvested as described in Wagner et al. 2017 [22].

2.2. Native Purification of KARI

The structural studies presented in this work have been obtained from the purification protocol described in Engilberge et al. 2019 (GlnA fraction, [23]). The preparation is shown in Figure 1A.

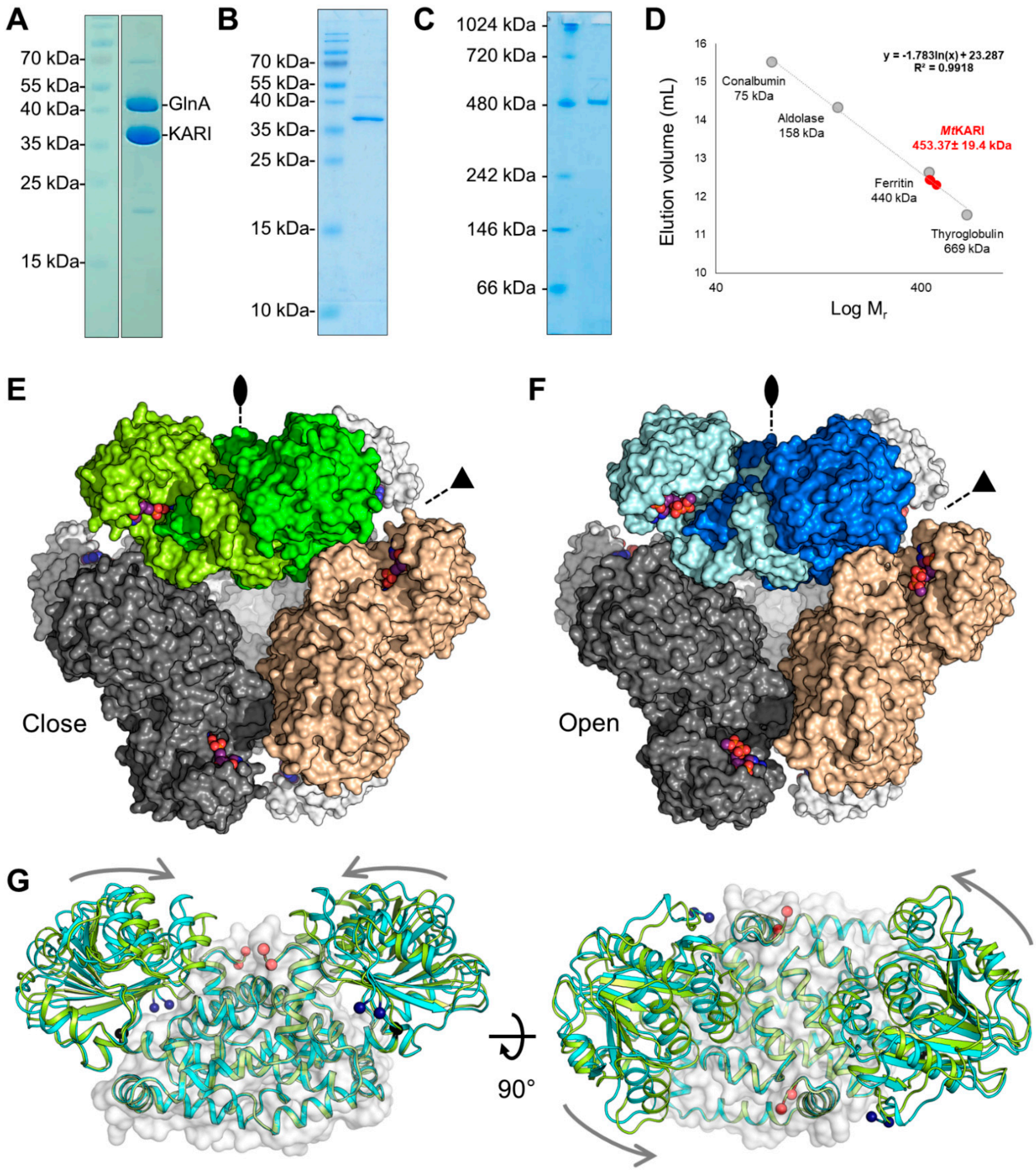

Figure 1. Purification and overall structure of *Mt*KARI. (**A**) SDS-PAGE of the sample used for crystallisation. (**B**) SDS and (**C**) hrCN-PAGE of enriched *Mt*KARI (2 µg) used for determination of the oligomeric state. (**D**) The dodecameric state of *Mt*KARI was determined by size exclusion chromatography (n = 3, see Figure S1). (**E**,**F**) Dodecameric structures of the closed (**E**, green surface) and open conformation (**F**, blue surface). Ligands are represented by balls and sticks with carbon, oxygen, nitrogen, phosphorus and Mg^{2+} coloured in purple, red, blue, orange and light green, respectively. (**G**) Superposition of the closed (green) and open (cyan) forms of *Mt*KARI represented as a cartoon. The common part (knot domain) is shown as a transparent surface. Blue and red balls indicate the positions of the N- and C-termini, respectively. The black balls indicate the hinge position. Grey arrows symbolise the closure movements.

An optimised purification procedure was used for the oligomerisation studies and is presented below.

Cell lysis was performed under a N_2/CO_2 (90:10%) atmosphere. 18 g (wet weight) of cells were thawed and diluted with ten volumes of 50 mM Tricine/NaOH pH 8.0, 2 mM dithiothreitol (DTT, Lysis Buffer), sonicated (5 × 10 s at 60% power, probe KE76 Bandelin SONOPULS Berlin, Germany) and centrifuged 45 min at 45,000× *g* at 18 °C. The supernatant was transferred to an anaerobic Coy tent containing a N_2/H_2 (97:3%) atmosphere, filtered through a 0.2 µm filter (Sartorius, Göttingen, Germany), and loaded on 20 mL HiTrap DEAE-Sepharose fast flow column (GE Healthcare, Munich, Germany). Elution was performed with a 0 to 500 mM NaCl linear gradient for 11.25 column volumes (CV) at a 2.5 mL/min flow rate. The sample of interest eluted between 175 and 240 mM NaCl. The resulting pool was diluted with two volumes of lysis buffer, filtered and loaded on 15 mL HiTrap Q-Sepharose high performance column (GE Healthcare, Munich, Germany). Elution was performed with a 250 to 400 mM NaCl linear gradient for 6 CV at a 2 mL/min flow rate. The fraction of interest eluted between 300 and 375 mM NaCl. The resulting pool was diluted with 3 volumes of 20 mM sodium phosphate buffer, pH 7.6, 2 mM DTT. The sample was filtered through 0.2 µm and injected on 10 mL Hydroxyapatite CHT Type 1 column (Bio-Rad Laboratories, Munich, Germany). The protein was eluted with a 20 to 200 mM sodium phosphate linear gradient for 4.5 CV at a 1.5 mL/min flow rate. The KARI and glutamine synthetase (GlnA [23]) eluted at a phosphate concentration between 75 and 200 mM. The fractions of interest were pooled and diluted with 1 volume of 25 mM Tris/HCl pH 7.6, 2 M $(NH_4)_2SO_4$ and 2 mM DTT, filtered through a 0.2 µm filter (Sartorius, Göttingen, Germany) and injected on a 5 mL HiTrap Phenyl-sepharose high performance column (GE Healthcare, Munich, Germany). The column was washed with 1.2 M $(NH_4)_2SO_4$ and the sample was eluted with a 1.2 to 0.8 M linear gradient of $(NH_4)_2SO_4$ for 12 CV followed by a 0.8 to 0 M linear gradient of $(NH_4)_2SO_4$ for 6 CV at a 1 mL/min flow rate. GlnA eluted between 0.70 and 0.52 M $(NH_4)_2SO_4$ while KARI was eluted between 0.52 and 0.42 mM $(NH_4)_2SO_4$. After concentration up to 300 µL with a 30-kDa cut-off centrifugation filter (Merck, Darmstadt, Germany), contaminants of KARI were separated by size-exclusion chromatography on a Superdex 200 Increase 10/300 GL (GE Healthcare, Munich, Germany) in 25 mM Tris/HCl pH 7.6, 2 mM DTT, 10% glycerol. The final pool was concentrated with a 30-kDa cut-off centrifugation filter (Merck, Darmstadt, Germany) to a final concentration of 14 g/L, as estimated by the Bradford method (Bio-Rad Laboratories, Munich, Germany). Each purification step was systematically controlled by denaturing sodium dodecyl sulphate polyacrylamide gel electrophoresis (SDS-PAGE).

2.3. High-Resolution Clear Native (hrCN) PAGE and Gel Filtration Experiments

The hrCN PAGE protocol was adapted from Lemaire et al. (2018) [24] Glycerol was added to the sample at a final amount of 20% *v*/*v*. Ponceau S at a final concentration of 0.001% *w*/*v* served as a marker to follow the migration. The buffer composition for the electrophoresis cathode was the following: 50 mM Tricine, 15 mM Bis-Tris/HCl, pH 7, 0.05% *w*/*v* sodium deoxycholate and 0.01% *w*/*v* dodecyl maltoside, while the anode buffer contained 50 mM Bis-Tris/HCl buffer pH 7. A 5 to 15% linear polyacrylamide gradient gel was used and electrophoresis was run with a constant 40 mA current (PowerPac^{TM} Basic Power Supply, Bio-Rad). After electrophoresis, protein bands were visualised with Ready Blue^{TM} Protein Gel stain (Sigma Aldrich, Hamburg, Germany). The native protein ladder used is NativeMark^{TM} Unstained Protein Standard (ThermoFischer Scientific, Dreieich, Germany).

Determination of the oligomeric state by gel filtration was performed on a Superose6 Increase 10/300 GL (GE Healthcare, Munich, Germany) in 25 mM Tris/HCl pH 7.4, 2 mM DTT, 10% glycerol at a 0.4 mL/min flow rate. High Molecular Weight range Gel Filtration Calibration Kit (GE Healthcare, Munich, Germany) was used as the protein standard.

2.4. Mass Spectrometry Protein Identification

Proteins were identified by Matrix Assisted Laser Desorption Ionization–Time of Flight Mass Spectrometry (MALDI-TOF-MS). Each gel band was cut out, chopped into small pieces and destained with 30% isopropanol (*v*/*v*) containing 50 mM NH_4HCO_3 and 30 mM thioglycolic acid (adjusted at pH 8.2), dehydrated with 100% isopropanol and was dried. Gel pieces were rehydrated in 5 mM NH_4HCO_3 and 8 mM DTT in 10% acetonitrile (*v*/*v*) containing 0.0025 g/L sequencing-grade modified trypsin (Promega, Madison, WI, USA) and incubated for 5 h at 22 °C. 1 µL of each supernatant was mixed with 1 µL solution of 3 mg/mL alpha-cyano-4-hydroxycinnamic acid in 80% acetonitrile (*v*/*v*) containing 0.3% trifluoroacetic acid onto a MALDI plate. The dried spots were measured automatically for MS and MS/MS in MALDI (4800 Proteomics Analyser, MDS Sciex, Concord, ON, Canada). Data were searched against an in-house database using a Mascot embedded into GPS explorer software (MDS Sciex).

2.5. Crystallisation

Crystallisation was performed aerobically by initial screening at 18 °C using the sitting drop method on 96-Well MRC 2-Drop polystyrene Crystallisation Plates (SWISSCI). The reservoir chamber was filled with 90 µL of crystallisation condition and the crystallisation drop was formed by spotting 0.7 µL protein with 0.7 µL of precipitant. For the close state structure, *Mt*KARI was crystallised at 13 mg/mL in a solution containing 20% (*w*/*v*) PEG 6000, 100 mM Tris/HCl pH 8.0 and 200 mM lithium chloride (Condition A). Transparent plates appeared after one week. For the open state, KARI was crystallised at 13.5 mg/mL in a solution containing 33% (*w*/*v*) PEG 5000 MME, 100 mM MES/NaOH pH 6.5 and 200 mM ammonium sulphate (condition B). Flower-shaped plates appeared after several weeks.

For the close state, KARI crystals obtained in condition A were soaked in the crystallisation solution supplemented with 30% *v*/*v* ethylene glycol for few seconds before freezing in liquid nitrogen. For the open state, crystals obtained in condition B were firstly soaked in the crystallisation solution supplemented with 20 mM NADP, 50 mM L-Tartrate and 50 mM $MgCl_2$ for 3 min. It must be noted that signs of aggregation were visible in the component soaking mixture. The crystals were further soaked in the crystallisation solution supplemented with 30% *v*/*v* glycerol before freezing.

2.6. X-ray Data Collection and Model Refinement/Validation

All diffraction experiments were performed at 100 K on PXII-X10SA beamline, Swiss Light Source synchrotron, Villigen, Switzerland. The data were processed with *XDS* and scaled with *SCALA* from the *CCP4* package [25]. The close state structure was solved by molecular replacement with *PHENIX* [26] using KARI from *Alicyclobacillus acidocaldarius* (PDB 4TSK, close state [15,17]) as a template. The open state model was also solved by molecular replacement with *PHENIX* by using the close state as template. Both models were manually built with *COOT* [27] and were refined with *PHENIX* by applying a twin refinement (intensity based) with the operator -l,-k,-h. Refinement with translational-liberation screw (TLS) was done during the last round for both models. The models were validated by the MolProbity server [28] (http://molprobity.biochem.duke.edu) (accessed on 26 September 2021). All figures were generated and rendered with PyMOL (Version 1.8, Schrödinger, LLC, New York, NY, USA).

2.7. Sequences Alignment and Phylogenetic Analysis

Protein sequences of *Mt*KARI and 13 other enzymes were used. The Figure 2D was designed with ESPript 3 [29], by using an alignment constructed with Clustal Omega [30]. The minimum Evolution Tree used for phylogenetic analysis was generated with the program MEGA [31]. A total of 2000 replicates were used to calculate each node score.

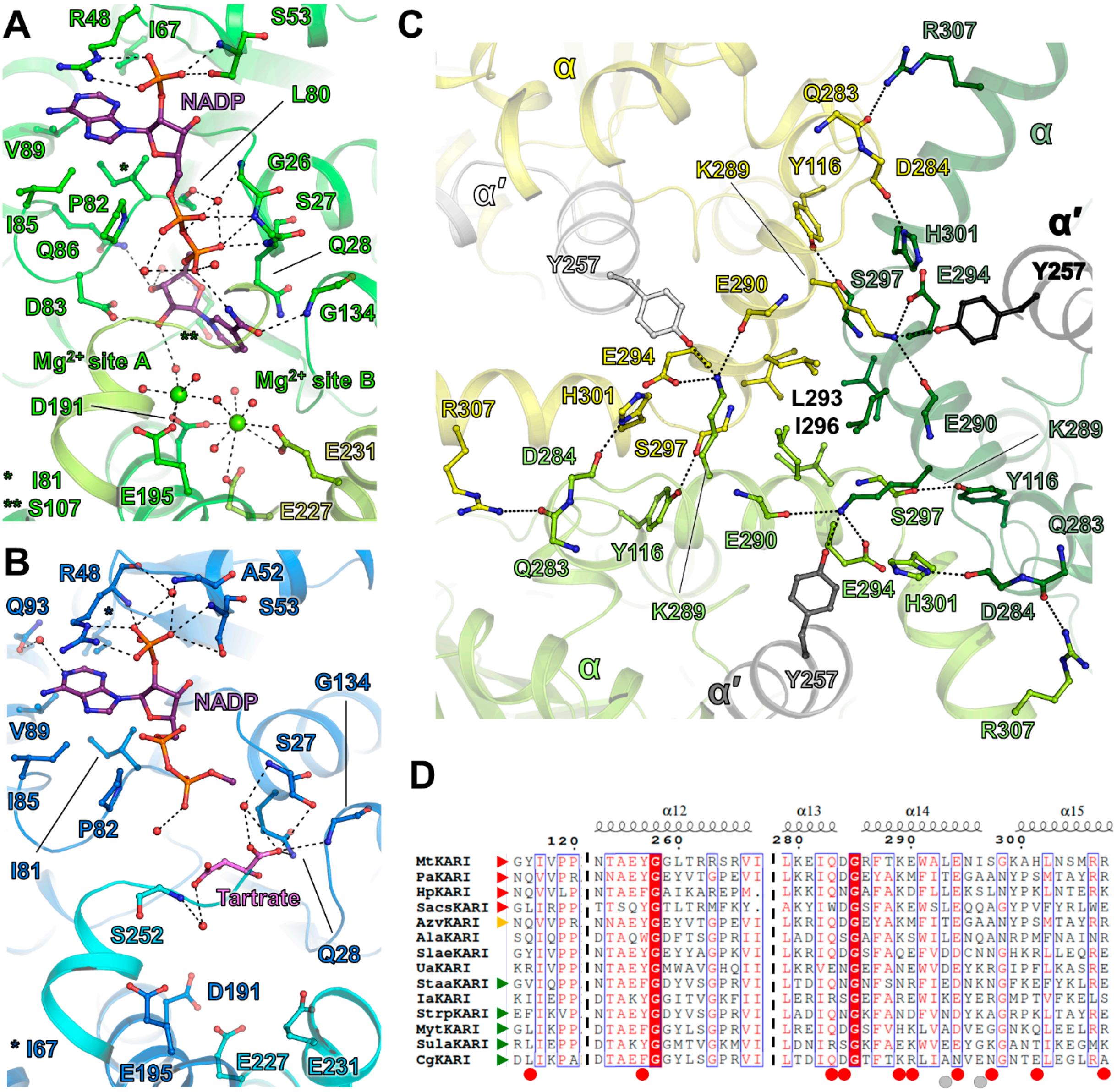

Figure 2. Ligand coordination and dodecamer stabilisation. The close (**A**) and open (**B**) state structures are represented in cartoon and are coloured in green and blue, respectively. The dimer chain is in a lighter colour. Ligands are represented as balls and sticks and are coloured in red, blue, orange and light green for oxygen, nitrogen, phosphorus and Mg^{2+}, respectively. NADP is coloured in purple, tartrate in pink. (**C**) An inside view of the close up of the trimeric interface. The structures are represented in cartoon and are coloured per monomer. Residues stabilising the interface are represented by sticks and are coloured, as in (**A**) and (**B**). (**A**–**C**) Hydrogen bonds are shown by black dashed lines. (**D**) ESPript alignment of the residues 115–120, 253–267 and 279–307 (*Mt*KARI numbering) in the structurally characterised KARIs. Red and green arrows indicate experimentally proven dodecameric and dimeric enzymes, respectively. The orange arrow points to *Azv*KARI, which is supposed to be dodecameric, even if initially described as a dimer [12,17]. Red circles indicate residues involved in polar contacts at the trimeric interface. Grey circles indicate residues involved in the hydrophobic interaction.

3. Results

3.1. Purification and Structural Characterisation of the Open/Closed Conformation of MtKARI

*Mt*KARI was initially purified by serendipity as a contaminant of the glutamine synthetase (GlnA) from cells grown under chemolithotrophic conditions at 65 °C (Figure 1A) [23]. The protein was identified by MALDI-TOF-MS. This initial *Mt*GlnA/*Mt*KARI mixture was used for crystallisation and crystals of both proteins were obtained illustrating once more

the selective power of crystallisation. As the presence of GlnA in the sample preparation was a hurdle for any further analysis, an optimised purification protocol was later used to separate *Mt*GlnA and *Mt*KARI, yielding a relatively pure *Mt*KARI sample (Figure 1B). hrCN-PAGE indicates a band at around 480 kDa, which is in accordance with the expected size of the dodecamer (438 kDa) and no band could be observed for the dimeric form (expected at 73-kDa, Figure 1C). A similar result is obtained by size-exclusion chromatography, the protein eluting as a unique peak at an elution volume compatible with a dodecameric state, independently of the concentration of the injected protein (Figures 1D and S1). The final yield of purified protein (1 mg) represents ~0.1% of the initial protein content extracted from the cells (1.2 g).

X-ray diffraction measurements were initially performed on crystals obtained from *Mt*KARI purified and crystallised without any additional ions or substrates. The crystalline form belonged to the *I*23 space group and the first structure was solved by molecular replacement using the closest structurally characterised homolog: the KARI from the *Firmicutes A. acidocaldarius* (PDB ID: 4TSK, *Ala*KARI, 56.53% identity [15,17]). The refinement and electron density quality was hampered by merohedral twinning, with a very high fraction (0.32, Table 1); however, the model was complete and was refined to a 2.1-Å resolution. The asymmetric unit contains a single monomer with the typical fold of a class I KARI (Figure S2): a Rossmann domain (2–183) and the knot domain (184–330). The cubic symmetry unveils a dodecameric oligomerisation (Figures 1E and S2C) in accordance with in solution experiments.

Table 1. X-ray crystallographic data and refinement statistics.

	***Mt*KARI Close State**	***Mt*KARI Open State**
**Data collection**		
Wavelength (Å)	1.73913	0.97916
Space group	*I*23	*I*23
Resolution (Å)	45.92—2.10 (2.21—2.10)	46.25—2.20 (2.32—2.20)
Cell dimensions: a = b = c (Å)	129.88	130.83
R_{merge} (%) [a]	9.5 (74.4)	16.3 (52.8)
R_{pim} (%) [a]	2.6 (21.2)	3.9 (16.6)
$CC_{1/2}$ [a]	0.999 (0.468)	0.998 (0.385)
I/σ_I [a]	18.3 (3.5)	23.7 (4.4)
Completeness [a]	100 (100)	100 (100)
Redundancy [a]	14.3 (13.3)	18.2 (10.9)
Nr. unique reflections [a]	21,408 (3,090)	19,073 (2766)
**Refinement**		
Resolution (Å)	41.07—2.10	41.37—2.20
Twinning fraction and operator	0.32 (-l,-k,-h)	0.12 (-l,-k,-h)
Number of reflections	21,408	19,073
R_{work}/R_{free} [b] (%)	17.59/18.86	18.64/21.23
Number of atoms		
Protein	2542	2536
Ligands/ions	96	49
Solvent	63	140
Mean B-value (Å^2)	45.9	30.3
Molprobity clashscore, all atoms	6.64	2.53
Ramachandran plot		
Favored regions (%)	96.32	97.23
Outlier regions (%)	0	0
Rmsd [c] bond lengths (Å)	0.009	0.006
rmsd [c] bond angles (°)	1.15	0.82
**PDB ID code**	7Q03	7Q07

[a] Values relative to the highest resolution shell are within parentheses. [b] R_{free} was calculated as the R_{work} for 5% of the reflections that were not included in the refinement. Refined models contained hydrogens. [c] rmsd, root mean square deviation.

The overall conformation of the enzyme fits the well-characterised close state. This conformation has the best fit with the structure of the KARI from *Slackia exigua* (*Slae*KARI), containing Mg^{2+} ions, NADPH and the substrate analogue L-tartrate (PDB ID: 4KQW [16], Figure S3, Table S1). In the obtained close state of *Mt*KARI, NADP and Mg^{2+} are bound in the structure, even though we did not add any ligands in the crystallisation and soaking solutions (Figures 2A and S4A,B). They therefore were natively co-purified from the cells of *M. thermolithotrophicus*.

We soaked crystals obtained from another crystallisation condition for a short time at a very high concentration of NADP, Mg^{2+} and L-tartrate, a substrate analogue already used in several studies [6,12,17]. X-ray diffraction data revealed a nearly identical cubic crystalline form, with a dilatation of the unit cell dimensions of 1-Å. The data analysis still revealed merohedral twinning, with a twin fraction of 0.12 and the model was refined to 2.2-Å resolution. While the dodecameric organisation is similar compared to the close state structure (Figure 1F), the Rossmann domain performed a 12° rotation between the two structures (Figures Figure 1G and S2D), which indicated that the soaked crystal is in an open conformation. Interestingly, while the presence of NADP and tartrate is visible in the electron density, Mg^{2+} ions seem to be absent in this structure, which yields the first structure of a Mg^{2+}-free NADP containing KARI (Figures Figure 2B and S4C,D). The best structural alignment is achieved with the structures of the Mg^{2+} and NADPH-containing KARIs from *Corynebacterium glutamicum* and *Streptococcus pneumoniae* (*Cg*KARI and *Strp*KARI, PDB IDs: 6JX2 [6] and 6L2I [11], respectively. Table S1). These structures, albeit containing ligands, are in an uncommon widely open conformation (Table S2, Figure S5). The structure of the open KARI of *Azotobacter vinelandii* (*Azv*KARI) containing only Mg^{2+} and Fe^{2+} fits better with the *Mt*KARI open state (PDB ID: 4XIY [17], Table S1, Figures S5 and S6).

3.2. Substrate Coordination in MtKARI Structures

The structure of *Mt*KARI in the close state harbours NADPH or $NADP^+$ (later mentioned as NADP for convenience) bound to the Rossmann domain, as well as two metals coordinated at the Mg^{2+} binding site in structurally characterised homologs (Figures 2A and S4A,B). Based on the closed conformation, the homology with other structures and their coordination, these ions were attributed as Mg^{2+}. While the NADP has a proper fit in the electron density, the suspected Mg^{2+} ions have a lower occupancy, especially for the second site (Figure S4A,B), which might come from the twinning or a rather loose hexacoordination for the second site (Figure S4B, site B). The first Mg^{2+} (site A) is hexacoordinated by Glu195, Asp191 and four waters, while the second ion (site B) is coordinated by Asp191, Glu231 and four water molecules with a far distant interaction with Glu227. The hexacoordination of site B is not octahedral and might be explained by a partially vacant site. In the elongated electron density bound to the two Mg^{2+} ions, a water network was modelled, but the possibility of another bound molecule cannot be excluded.

According to the classification and nomenclature from Cahn and colleagues (2015) [17], *Mt*KARI exhibits a classical 7-residue long β2αB selectivity loop (-LRPNGAS-) in the Rossmann domain corresponding to the stretch 47-53. The Arg48 and Ser53 selectively accommodate and stabilise the 2′-phosphate of NADP in the close state, similar to what is observed in *Slae*KARI [16,17]; however, the second stabilising serine (Ser61 in *Slae*KARI) does not exist in *Mt*KARI (Figure S7). A hydrophobic pocket contains the adenine moiety, several hydrogen bonds bind the pyrophosphate to the protein backbone and the ribose-nicotinamide part is bound by Asp83, Gly134 and a water network. A similar binding mode was observed in *Ala*KARI, albeit exhibiting a shortened 6-residues β2αB selectivity loop (Figure S7). In contrast, the open state shows a rather loose binding, in which only the 2-phospho-adenosine part is anchored, while the pyrophosphate shows no direct contacts with the protein (Figure 2B). However, because of the absence of Mg^{2+} stabilising a water network and because of the presence of tartrate at the expected nicotinamide position, the adenine-nicotinamide part of NADP undergoes a drastic movement and protrudes out of the cavity, becoming too flexible to be modelled after the pyrophosphate group. The

substrate analogue tartrate is not found at the Mg^{2+} ions vicinity as described in the other structures of KARI (see *Ala*KARI in Figure S7). In the artefactual Mg^{2+}-free structure of *Mt*KARI, tartrate takes the vacant position of the nicotinamide group of NADPH and is loosely bound through its carboxy-groups by a hydrogen bond network provided by Ser27, Gln28, Gly134 and water molecules (Figures 2B and S4C,D).

3.3. A Stable Dodecameric State Is Maintained by a Few Anchoring Residues

As stated above, native electrophoresis and size exclusion chromatography show that *Mt*KARI is a stable dodecameric enzyme (Figures 1C–F and S1). The dodecamer consists of six dimer units positioned on the tetrahedron edges and the trimeric assembly is localized at the four vertices, as previously pointed out by Lv and colleagues (2016) [12]. The crystal structures presented in this work highlight specific residues that should be involved in this assembly. The interaction is maintained by hydrogen bonds and hydrophobic interactions, organised by strata and principally involving the 286–298 α-helix (Figure 2C). Facing the interior of the dodecamer, Lys289 protrudes from the 286–298 α-helix and connects with the opposite KARI dimer (αα′), forming hydrogen bonds with αGlu290, αGlu294 and interacting with α′Tyr257 (Figure 2C). Lysine and tyrosine residues were already highlighted as putative main actors of the dodecamerisation [12]. As each KARI dimer forms the same interaction, a trimeric organisation is formed, with hydrogen bonds between αHis301-αAsp284, αArg307-αGln283 and αTyr116-αSer297 stabilising the dimer–dimer interaction area. Furthermore, at the outside of the trimeric interface, facing the external solvent, the hydrophobic residues Leu293 and Ile296 form a compact hydrophobic environment, which locks the trimer (Figure 2C).

The sequence alignment of all structurally characterised KARIs suggests that none of the above-mentioned residues could be selected as a hallmark for dodecamerisation, because they are either not conserved in the dodecameric enzymes, or are not specific to them (Figure 2D, Table S3). The trimeric interface differs in other enzymes, yet they conserve some interaction networks (Lys-Tyr-Glu, Figure S8). The *Pa*KARI (*Pseudomonas aeruginosa* [7]) exhibits a different, rather simplified, trimeric interface compared to *Mt*KARI and *Sacs*KARI (*Saccharolobus solfataricus,* formerly *Sulfolobus solfataricus* [13]), which might be linked to the thermophilic nature of *M. thermolithotrophicus* and *S. solfataricus*. The phylogenic analysis performed on structurally characterised KARI and the dodecameric KARI from *Helicobacter pylori* [7] hints that dodecameric KARIs are not a monophyletic group (Figure S9). These results suggest that oligomeric state transitions could have occurred separately in archaea and bacteria.

4. Discussion

Ketol-acid reductoisomerase catalyses the second step of the branched-chain amino acid synthesis, a complicated two-step reaction that requires a high specificity for its substrates and coenzyme. As a key actor of this pathway found in prokaryotes and plants, KARI receives increasing attention as a potential drug target, but also for industrial applications for amino acid production. Chemolithoautotrophic methanogens, such as *M. thermolithotrophicus*, depend on this enzyme to synthesise their branched-chain amino acids. The dodecameric structure of KARI from *M. thermolithotrophicus* was obtained in both closed and open conformations, illustrating the natural induced fit movements preceding catalysis. The purified and crystallised native closed enzyme harbours Mg^{2+} ions and NADP, which shows the coenzyme selectivity. This specificity is in accordance with its amino acid sequence and the 2′-phosphate coordination in the structure. The open form exhibits a Mg^{2+}-free/NADP bound state, leading to a mobile adenine–nicotinamide moiety. Since the crystallisation condition was different between the two states, we cannot rule out that the Mg^{2+} sites were already depleted in the crystals prior to the soak. For instance, the crystallisation condition could have an effect on the positioning of Asp191, Glu195, Glu227 and Glu231. The high concentration of NADP and tartrate used for soaking might have also destabilised the metal binding site and aborted the correct positioning of

the nicotinamide moiety. This Mg^{2+}-depleted structure is catalytically inactive, since the substrate/coenzyme binding and initial alkyl migration that occurs in the reaction depends on these Mg^{2+} [32]. Nevertheless, despite being artificial, this structural snapshot could provide new leads for in silico drug design approaches to lock the enzyme in an open and inactive state.

As has already been pointed out by other structural studies of class I KARIs, the overall organisation of the enzyme is remarkably conserved [12,13,17]. Structures of KARI from archaea and bacteria are superposable, and share the conformational movement upon ligand binding, and coordinate their ligands with equivalent, if not identical, residues. This reflects the selective pressure on this enzyme, which is coherent for its necessity in the absence of branched-chain amino acid resources. The numerous available structures and biochemical data [6–8,10–13,15–20] indicate that the main variable among KARIs are the cofactor (NADH/NADPH) selectivity and the oligomeric state.

The oligomeric state of KARIs is intriguing, as it appears not to exhibit a specific pattern. Dimeric and dodecameric enzymes were described in both bacteria and archaea, and depend on the phyla. For example, with the current knowledge gathered, it seems that proteobacteria (*H. pylori*, *P. aeruginosa* and *A. vinelandii*) encode dodecameric KARIs, while Gram-positive bacteria (*S. aureus*, *S. pneumoniae*, *M. tuberculosis*, *C. glutamicum*, *A. acidocaldarius*) encode dimeric KARIs. An effect of heterologous production or tag hindrance can be ruled out, since dimeric and dodecameric KARIs were successfully produced in *E. coli*. Experimental artefacts or a dodecamer/dimer equilibrium also have to be ruled out, since the oligomeric state of KARIs were confirmed by different techniques and experimental setups (Figures 1C,D and S1, Table S3). Structural studies interestingly revealed a similar diversity in class II KARIs, which could be either dimeric or tetrameric [14]. Residues apparently involved in the dodecamerisation of KARI belonging to class I have no conserved sequence motif related to the trimeric interface and the different characterised dodecamers are stabilised in slightly different manners. Since the trimeric interface is relatively conserved in terms of secondary elements and lengths, sequence analysis is not sufficient to predict quaternary organisation, and biochemical characterisation must prevail in the future to confirm the oligomeric state of different KARIs. The phylogeny of a small set of sequences suggests that the dimeric and dodecameric KARIs do not belong to divergent enzyme groups, but rather that a switch of oligomeric state occurred several times during archaeal and bacterial evolution.

Does the oligomeric state of KARI imply a selective pressure? This question cannot be answered, since the impact of the dodecamerisation on the enzymatic activity and metabolism has not been investigated yet to our knowledge. The oligomerisation is apparently not linked to the living temperature of the organisms, as dimeric enzymes were found in thermophiles (e.g., *S. acidocaldarius*) and mesophiles (e.g., *M. tuberculosis*), similarly to dodecameric KARIs (e.g., *M. thermolithotrophicus* and *P. aeruginosa*). The same conclusions can be reached regarding the salinity and pH of the culture media (Table S4), suggesting that the oligomeric state does not depend on the physicochemical conditions. It could be interesting to confirm whether the dodecamerisation affects substrate diffusion and binding kinetics, as well as metabolic fluxes, in vivo. Verification of this hypothesis could be achieved by the mutation of strategic residues, which are important for the oligomerisation (e.g., in *P. aeruginosa* Lys288 could be exchanged by a tryptophan to generate a sterical encumbrancy). These mutants would be used for the comparison of growth fitness or a competition experiment of a strain producing a dimeric or dodecameric version of the same KARI in a medium devoid of branched-chain amino acids (i.e., *P. aeruginosa*). Such results could have tremendous repercussions on the microbial branched-chain amino acid yields in biotechnological processes.

Supplementary Materials: The following are available online at https://www.mdpi.com/article/10.3390/biom11111679/s1, Figure S1: Size exclusion chromatography of *Mt*KARI at different protein concentrations; Figure S2: Domain organisation and assembly; Figure S3: Superposition of the structures of close *Mt*KARI and *Slae*KARI (4KQW); Figure S4: Electron density and omit map of

Mg^{2+}, NADP, and tartrate in *Mt*KARI structures; Figure S5: Structural alignment of open and close state KARI structures; Figure S6: Superposition of the structures of open *Mt*KARI and *Azv*KARI (4XIY); Figure S7: Colocalisation of NADP and Mg^{2+} in close state structures of *Mt*KARI, *Ala*KARI (4TSK) and *Slae*KARI (4KQW); Figure S8: Trimeric interface in *Pa*KARI (1NP3) and *Sacs*KARI (6KPJ); Figure S9: Evolutionary relationships of the characterised KARIs used in this work; Table S1: Structural alignment of the *Mt*KARI open and close states to the structurally characterised non-mutant KARIs; Table S2: Structural alignment of the structure of KARIs from C. glutamicum and S. pneumoniae to the structurally characterised non-mutant KARIs; Table S3: Oligomeric state of KARI; Table S4: Oligomeric state of KARIs depending on optimal growing conditions of the organisms.

Author Contributions: Protein extraction and purification was performed by T.W. and M.-C.M. Phylogenetic analyses, electrophoreses and size exclusion chromatography were performed by O.N.L. Crystallisation, X-ray data collection, processing was performed by T.W. Model building, refinement and structural analyses were performed by O.N.L. and T.W. Mass spectrometry identification was done by J.K. The data were interpreted and the paper was written by O.N.L. and T.W. with the contributions and final approval of all co-authors. All authors have read and agreed to the published version of the manuscript.

Funding: The research was funded by the Max-Planck Gesellschaft.

Institutional Review Board Statement: Not applicable.

Data Availability Statement: The structures were deposited in the protein data bank under the ID: 7Q03 (close form) and 7Q07 (open form).

Acknowledgments: We thank the Max Planck Institute for Marine Microbiology and the Max-Planck-Society for continuous support. We particularly thank Seigo Shima for his support in the initial stage of this project performed at the Max Planck Institute for Terrestrial microbiology and his critical correction of the manuscript. We acknowledge the SLS synchrotron for beam time allocation and the beamline staff for assistance with data collection. Many thanks are going to Ulrich Ermler and the Max Planck Institute for Biophysics (Frankfurt) to provide us with SLS beam time. We are also thankful to Christina Probian, Ramona Appel and Mélissa Belhamri for their continuous support in the Microbial Metabolism laboratory.

Conflicts of Interest: The authors declare no conflict of interest.

References

1. Dumas, R.; Biou, V.; Halgand, F.; Douce, R.; Duggleby, R.G. Enzymology, Structure, and Dynamics of Acetohydroxy Acid Isomeroreductase. *Acc. Chem. Res.* **2001**, *34*, 399–408. [CrossRef]
2. Bayaraa, T.; Kurz, J.L.; Patel, K.M.; Hussein, W.M.; Bilyj, J.K.; West, N.P.; Schenk, G.; McGeary, R.P.; Guddat, L.W. Discovery, Synthesis and Evaluation of a Ketol-Acid Reductoisomerase Inhibitor. *Chemistry* **2020**, *26*, 8958–8968. [CrossRef]
3. Yu, M.J.; Wu, J.; Chen, S.L. Mechanism and Inhibitor Exploration with Binuclear Mg Ketol-Acid Reductoisomerase: Targeting the Biosynthetic Pathway of Branched-Chain Amino Acids. *ChemBioChem* **2020**, *21*, 381–391. [CrossRef]
4. Kandale, A.; Patel, K.; Hussein, W.M.; Wun, S.J.; Zheng, S.; Tan, L.; West, N.P.; Schenk, G.; Guddat, L.W.; McGeary, R.P. Analogues of the Herbicide, N-Hydroxy-N-isopropyloxamate, Inhibit *Mycobacterium tuberculosis* Ketol-Acid Reductoisomerase and Their Prodrugs Are Promising Anti-TB Drug Leads. *J. Med. Chem.* **2021**, *64*, 1670–1684. [CrossRef]
5. Wang, Y.Y.; Zhang, F.; Xu, J.Z.; Zhang, W.G.; Chen, X.L.; Liu, L.M. Improvement of L-Leucine Production in *Corynebacterium glutamicum* by Altering the Redox Flux. *Int. J. Mol. Sci.* **2019**, *20*, 2020. [CrossRef]
6. Lee, D.; Hong, J.; Kim, K.J. Crystal Structure and Biochemical Characterization of Ketol-Acid Reductoisomerase from *Corynebacterium glutamicum*. *J. Agric. Food Chem.* **2019**, *67*, 8527–8535. [CrossRef]
7. Ahn, H.J.; Eom, S.J.; Yoon, H.J.; Lee, B.I.; Cho, H.; Suh, S.W. Crystal structure of class I acetohydroxy acid isomeroreductase from *Pseudomonas aeruginosa*. *J. Mol. Biol.* **2003**, *328*, 505–515. [CrossRef]
8. Chen, C.-Y.; Ko, T.-P.; Lin, K.-F.; Lin, B.-L.; Huang, C.-H.; Chiang, C.-H.; Horng, J.-C. NADH/NADPH bi-cofactor-utilizing and thermoactive ketol-acid reductoisomerase from *Sulfolobus acidocaldarius*. *Sci. Rep.* **2018**, *8*, 7176. [CrossRef] [PubMed]
9. Biou, V.; Dumas, R.; Cohen-Addad, C.; Douce, R.; Job, D.; Pebay-Peyroula, E. The crystal structure of plant acetohydroxy acid isomeroreductase complexed with NADPH, two magnesium ions and a herbicidal transition state analog determined at 1.65 A resolution. *EMBO J.* **1997**, *16*, 3405–3415. [CrossRef] [PubMed]
10. Patel, K.M.; Teran, D.; Zheng, S.; Kandale, A.; Garcia, M.; Lv, Y.; Schembri, M.A.; McGeary, R.P.; Schenk, G.; Guddat, L.W. Crystal Structures of *Staphylococcus aureus* Ketol-Acid Reductoisomerase in Complex with Two Transition State Analogues that Have Biocidal Activity. *Chemistry* **2017**, *23*, 18289–18295. [CrossRef]

11. Kim, G.; Shin, D.; Lee, S.; Yun, J.; Lee, S. Crystal Structure of IlvC, a Ketol-Acid Reductoisomerase, from *Streptococcus pneumoniae*. *Crystals* **2019**, *9*, 551. [CrossRef]
12. Lv, Y.; Kandale, A.; Wun, S.J.; McGeary, R.P.; Williams, S.J.; Kobe, B.; Sieber, V.; Schembri, M.A.; Schenk, G.; Guddat, L.W. Crystal structure of *Mycobacterium tuberculosis* ketol-acid reductoisomerase at 1.0 Å resolution—A potential target for anti-tuberculosis drug discovery. *FEBS J.* **2016**, *283*, 1184–1196. [CrossRef] [PubMed]
13. Chen, C.Y.; Chang, Y.C.; Lin, B.L.; Lin, K.F.; Huang, C.H.; Hsieh, D.L.; Ko, T.P.; Tsai, M.D. Use of Cryo-EM To Uncover Structural Bases of pH Effect and Cofactor Bispecificity of Ketol-Acid Reductoisomerase. *J. Am. Chem. Soc.* **2019**, *141*, 6136–6140. [CrossRef]
14. Tyagi, R.; Duquerroy, S.; Navaza, J.; Guddat, L.W.; Duggleby, R.G. The crystal structure of a bacterial class II ketol-acid reductoisomerase: Domain conservation and evolution. *Protein Sci.* **2005**, *14*, 3089–3100. [CrossRef] [PubMed]
15. Brinkmann-Chen, S.; Cahn, J.K.B.; Arnold, F.H. Uncovering rare NADH-preferring ketol-acid reductoisomerases. *Metab. Eng.* **2014**, *26*, 17–22. [CrossRef]
16. Brinkmann-Chen, S.; Flock, T.; Cahn, J.K.B.; Snow, C.D.; Brustad, E.M.; McIntosh, J.A.; Meinhold, P.; Zhang, L.; Arnold, F.H. General approach to reversing ketol-acid reductoisomerase cofactor dependence from NADPH to NADH. *Proc. Natl. Acad. Sci. USA* **2013**, *110*, 10946–10951. [CrossRef] [PubMed]
17. Cahn, J.K.B.; Brinkmann-Chen, S.; Spatzal, T.; Wiig, J.A.; Buller, A.R.; Einsle, O.; Hu, Y.; Ribbe, M.W.; Arnold, F.H. Cofactor specificity motifs and the induced fit mechanism in class I ketol-acid reductoisomerases. *Biochem. J.* **2015**, *468*, 475–484. [CrossRef]
18. Cahn, J.K.B.; Baumschlager, A.; Brinkmann-Chen, S.; Arnold, F.H. Mutations in adenine-binding pockets enhance catalytic properties of NAD(P)H-dependent enzymes. *Protein Eng. Des. Sel.* **2016**, *29*, 31–38. [CrossRef]
19. Cahn, J.K.B.; Werlang, C.A.; Baumschlager, A.; Brinkmann-Chen, S.; Mayo, S.L.; Arnold, F.H. A General Tool for Engineering the NAD/NADP Cofactor Preference of Oxidoreductases. *ACS Synth. Biol.* **2017**, *6*, 326–333. [CrossRef]
20. Chen, C.-Y.; Chang, Y.-C.; Lin, B.-L.; Huang, C.-H.; Tsai, M.-D. Temperature-Resolved Cryo-EM Uncovers Structural Bases of Temperature-Dependent Enzyme Functions. *J. Am. Chem. Soc.* **2019**, *141*, 19983–19987. [CrossRef]
21. Huber, H.; Thomm, M.; König, H.; Thies, G.; Stetter, K.O. *Methanococcus thermolithotrophicus*, a novel thermophilic lithotrophic methanogen. *Arch. Microbiol.* **1982**, *132*, 47–50. [CrossRef]
22. Wagner, T.; Koch, J.; Ermler, U.; Shima, S. Methanogenic heterodisulfide reductase (HdrABC-MvhAGD) uses two noncubane [4Fe-4S] clusters for reduction. *Science* **2017**, *357*, 699–703. [CrossRef]
23. Engilberge, S.; Wagner, T.; Santoni, G.; Breyton, C.; Shima, S.; Franzetti, B.; Riobé, F.; Maury, O.; Girard, E. Protein crystal structure determination with the crystallophore, a nucleating and phasing agent. *J. Appl. Cryst.* **2019**, *52*, 722–731. [CrossRef]
24. Lemaire, O.N.; Infossi, P.; Ali Chaouche, A.; Espinosa, L.; Leimkühler, S.; Giudici-Orticoni, M.T.; Méjean, V.; Iobbi-Nivol, C. Small membranous proteins of the TorE/NapE family, crutches for cognate respiratory systems in *Proteobacteria*. *Sci. Rep.* **2018**, *8*, 13576. [CrossRef]
25. Winn, M.D.; Ballard, C.C.; Cowtan, K.D.; Dodson, E.J.; Emsley, P.; Evans, P.R.; Keegan, R.M.; Krissinel, E.B.; Leslie, A.G.; McCoy, A.; et al. Overview of the *CCP4* suite and current developments. *Acta Cryst. D Biol. Cryst.* **2011**, *67*, 235–242. [CrossRef]
26. Liebschner, D.; Afonine, P.V.; Baker, M.L.; Bunkóczi, G.; Chen, V.B.; Croll, T.I.; Hintze, B.; Hung, L.W.; Jain, S.; McCoy, A.J.; et al. Macromolecular structure determination using X-rays, neutrons and electrons: Recent developments in *Phenix*. *Acta Cryst. D Struct. Biol.* **2019**, *75*, 861–877. [CrossRef]
27. Emsley, P.; Lohkamp, B.; Scott, W.G.; Cowtan, K. Features and development of *Coot*. *Acta Cryst. D Biol. Cryst.* **2010**, *66*, 486–501. [CrossRef] [PubMed]
28. Chen, V.B.; Arendall, W.B., 3rd; Headd, J.J.; Keedy, D.A.; Immormino, R.M.; Kapral, G.J.; Murray, L.W.; Richardson, J.S.; Richardson, D.C. *MolProbity*: All-atom structure validation for macromolecular crystallography. *Acta Cryst. D Biol. Cryst.* **2010**, *66*, 12–21. [CrossRef] [PubMed]
29. Robert, X.; Gouet, P. Deciphering key features in protein structures with the new ENDscript server. *Nucleic Acids Res.* **2014**, *42*, W320–W324. [CrossRef]
30. Madeira, F.; Park, Y.M.; Lee, J.; Buso, N.; Gur, T.; Madhusoodanan, N.; Basutkar, P.; Tivey, A.R.N.; Potter, S.C.; Finn, R.D.; et al. The EMBL-EBI search and sequence analysis tools APIs in 2019. *Nucleic Acids Res.* **2019**, *47*, W636–W641. [CrossRef] [PubMed]
31. Kumar, S.; Stecher, G.; Li, M.; Knyaz, C.; Tamura, K. *MEGA X*: Molecular Evolutionary Genetics Analysis across Computing Platforms. *Mol. Biol. Evol.* **2018**, *35*, 1547–1549. [CrossRef] [PubMed]
32. Chunduru, S.K.; Mrachko, G.T.; Calvo, K.C. Mechanism of ketol acid reductoisomerase. Steady-state analysis and metal ion requirement. *Biochemistry* **1989**, *28*, 486–493. [CrossRef] [PubMed]
