# Supplementary material for: Picoplankton nitrogen guilds in the tropical and subtropical oceans: From the surface to the deep
Source: PLoS One. 2025 Nov 5;20(11):e0335222. doi: 10.1371/journal.pone.0335222 (PMC12588538; doi:10.1371/journal.pone.0335222)
Supplement: Attachment — Submitted filename: Response to Reviewers.docx [file pone.0335222.s002.docx]

**Additional Editor Comments:**

It is important to note the differences between the 2 reviewers reflecting their different backgrounds.  Because you are addressing a subject crossing discipline boundaries it is important to make your writing clear to all disciplines.  The biodiversity debate is an obvious example of this and including some references as to the utility of an index that includes both abundance and species richness is probably needed if you think that using that rather than abundance and richness separately brings more to the discussion. Other than that- they both offer good points about making the manuscript clearer.

We have made an effort to clarify our reasoning as well as our procedures. The comments of the two reviewers were helpful identifying where more work was needed. With respect to the diversity issue, we provide what we hope is a clear explanation (see response to ref 2). Our way of combing abundance and diversity provides a very efficient and visually easy way to interpret presentation of a rather complex data set

Our response in blue.

5. Review Comments to the Author

Please use the space provided to explain your answers to the questions above. You may also include additional comments for the author, including concerns about dual publication, research ethics, or publication ethics. (Please upload your review as an attachment if it exceeds 20,000 characters)

Reviewer #1: Rivas-Santisteban et al.,’s paper approaches the distribution of picoplankton (mostly bacteria) in the ocean from the perspective of ecological guilds, defined as lists of organisms exploiting the same resource (in this case nitrogen) in the same way regardless of their phylogenetic relatedness. This approach, rarely seen in biological oceanography but very interesting, is explored using the Malaspina dataset, with 11 CTD casts across the tropical oceans ranging from the epipelagic to the bathypelagic realm. I enjoyed reading this paper and, to my knowledge, this could be a valuable piece of work for many oceanographers who are not familiar with the ecological guild approach. However, I have a few concerns about the oceanographic interpretation of the results that I think could be improved (and I hope my suggestions are somehow helpful) and some writing suggestions to make the paper more readable and impactful.

We thank the reviewer’s acknowledgement of the value of the method employed, and his/her constructive attitude.

Major points

Vertical and horizontal mapping - A strong point of this piece of work is the vertical extent and the geographic variability. Figure 1) is however quite confusing. For example: what do the colours of the dots in Figure 1a mean? I would suggest removing the equator line and the text on the figure (which is not very helpful) and instead include only the circles for the used stations (ideally connected by the shiptrack).

Each color meant a different sampling station. However, the reviewer is right: it was quite unnecessary. Now, we have black filled circles for Malaspina stations not used in the present work, white dots for those used here and special colors for the three stations analyzed in detail. We also use a new map without copyright issues, removed the equator line and text in the figure. We have not added the cruise track but we think that, at this large scale, the succession of stations should be enough.

Station 120 seems a long way away from the Mexico coast and there is another station that looks closer so I recommend checking that there are no mistakes there.

Station 141 is marked as North Atlantic but it’s clearly in the South Atlantic (is that the SAtl station mentioned in the text?).

We thank the reviewer for pointing this out. In fact, the vectorial layer moved accidentally and both stations 120 and 141 were off-position. Station 120 is ~733 km southwest of Acapulco, Mexico (10.759, -102.442). We hope we will not have this problem again

I also think that a crucial piece of information is the distribution of depths of the samples. The bathypelagic is way thicker than the other realms so is the density of the vertical sampling the same? This might also affect the interpretation of the diversity and relative importance of the marker genes (if the sample numbers are different for different realms).

Sampling depths can be seen en Figure 1B and with more precision in table S4. The three layers surely have different thicknesses. However, the meso- and bathypelagic have more uniform conditions than the epipelagic. Sampling depths were selected to retrieve changes in parameters more accurately, as is customary in oceanographic cruises.

With respect to the data treatment our normalization includes the number of samples per categorical depth. We consider the distribution of metagenomes in each layer to calculate the relative abundance. We now make this explicit in the main text.

I would also recommend to use Figure 1a at its best and ideally have the average oxygen distribution in the background since it’s something used to interpret the results. The data should be freely accessible from WOCE (https://woceatlas.ucsd.edu/) or there are some other model re-analyses that could help. I’m also a bit curiousby a few coastal stations (like those off South Africa and South Australia) - how do they relate to the average?

We played around with different ways to display oxygen data in our fig 1a, but oxygen distribution is highly dependent on depth and, in fact, the most interesting minimum in oxygen is found at different depths in different stations. In summary, we did not find a way to show the values in a 2D map. Thus, we have added the oxygen concentrations for each sample in a new supplementary figure S1.

North Atlantic station - The authors provide a reasonable explanation of why station 120 is different from most of the other locations, but I didn’t find the same level of detail for the North Atlantic station which still presents a very interesting pattern. I wonder if presenting in the supplementary information some profiles of temperature, salinity and oxygen can possibly help the interpretation?

We agree. In the already mentioned new supplementary figure S1, we included other variables along the vertical profiles.

More details are required for the GAM model - I think it is something that is really important in the logic of the paper but I struggled to understand. Is the predictive variable a categorical one? And are any interaction terms considered? For example how would the delta from a bathypelagic sample in the OMZ be treated?

We agree that this part of the paper is fundamental. After revision, we also agree with the reviewer that the clarity in the model part could be improved. We rephrased and improved the explanations given in sections 2.4 and 4.5.

However, we wanted to make clear that our analysis did not consist of a GAM model, which has smoothing functions, but of a series of independent GLMs, each of which assumes always a linear relationship between the categorical predictor and the response variable (delta in our case). This was made for each context: OMZ vs rest, Bathypelagic vs the rest, etc. We extracted the coefficients and showed them in Table 1. We considered this was the most conservative method, because categories were not truly independent. Thus, we didn’t considered interaction terms. But there is a justification for avoiding this analysis: our dataset comprises 75 metagenomes distributed across 11 stations and 3 depth layers. If we were to split the data by **Station × Depth**, many categories in the matrix would end with very few replicates (sometimes just one). This over parameterization may obscure the real effect exerted by the category.

Nevertheless, we provided a supplementary figure to capture specific departures like the one expressed by the reviewer (new fig. S6, old fig. S5). In that case (OMZ bathypelagic), we observe a positive effect on delta compared to the other oceanic layers at the OMZ.

Minor points
Line 99 - ‘as expected’ : why is that expected?

We have tried to improve the sentence.

Line 101 - were any other stations in OMZ? Perhaps having some oxygen distribution map on the background of figure 1 could be really helpful.

Data are shown in new figure S1. Although there were several stations showing oxygen minima, only station OMZ (120) reached concentrations below 60 µM (for a substantially thick water layer), which is usually taken as the threshold for an OMZ.

Line 176: ‘could be divided’ I suggest replacing this with ‘were divided’

Done.

Lines 283-288: This is an interesting interpretation, but to be plausible I think more detail should be provided: How frequent are oil spills in the area? And for how long do we expect them to impact the bacterial community? This paragraph also needs to be backed up with some references

We added a few lines and a reference indicating a relatively close area of natural oil seeps. But, this is only a hypothesis.

Line 311: I’d avoid using the term ‘general ocean’ as that could mean something different to different readers.

Replaced it with “ocean average”.

Section 4.1: Here I think more details about the bottle depths, etc need to be included.

We added supplementary table (S4) with this information.

Reviewer #2: In their study, Rivas-Santisteban and co-workers ask whether there are picoplankton “guilds” that are responsible for two aspects of nitrogen metabolism in the tropical and subtropical oceans – uptake (acquisition) and metabolism (redox). They do so by analyzing metagenomes collected on the Malaspina cruises from 75 depths, focusing on a selected set of genes representing these two types of processes. Additionally, they incorporate into their analyses not only the abundance of the different genes but also their diversity, reasoning that increased diversity also means that the functions (or the guilds performing them) are more robust to perturbations.

The questions asked by the authors are surely interesting, and, in principle, I think their analyses could answer these questions. I especially respect the care taken by the authors to make sure that the sequences they analyze are true orthologs, rather than paralogs (or just genes with a level of similarity above a specific cutoff). However, I found the manuscript extremely difficult to read and follow. Perhaps I do not have all of the ecological background needed to understand the manuscript, but the following examples can highlight some places where I found the manuscript to be very unclear. I therefore recommend it undergoes a major revision before being acceptable to PLoS One.

We thank the reviewer for their insights and constructive attitude towards the question and methods displayed in the paper. We have tried to improve the manuscript throughout

1) In the introduction, I struggled to understand what the authors mean by, for example, “functional response” (lines 37 and 38), “membership is not a static quality” (lines 42-43), what are the predictor variables and the functional profile (lines 43-44), and what is the “clear division between biomes and guilds” (line 93). It was also not clear what the research question was, whether such questions were asked before (e.g. as part of the set of papers from TARA oceans), and how the specific data and methodology would build upon previous knowledge.

We understand that our wording was not always clear enough. We have made the following changes:.

functional response. We added: observed microbial functional responses (i.e. appearance, depletion and diversification of biochemical functions)

Static quality, We added: That is to say, while ``species A'' will always be considered ``species A'' (static), function X can be performed only under certain conditions and species, which fluctuate (dynamic).

We further clarified the predictor variables in the introduction (depth and N:P inferred by location), and replaced “biomes” by “contexts”, referred to each combination of depth x location.

2) I also found the concept of “expected richness” difficult to follow, both in the materials and methods (lines 436-442), in the supplementary information (Figure S3), and in the results section (lines 159-174). While I understand in very broad terms that the diversity of sequences is expected to increase with their abundance, there must be many cases where this is not so clear.

As the reviewer mentions, the diversity of a gene increases with the abundance of the gene. We determined the expected diversity for each gene with a regression with its abundance as independent variable using all the data from the metagenomes (all the different variants of a gene found and their respective abundances). Then, we look for those cases where the diversity is higher or lower than this regression would predict. These are precisely the “many cases where this is not so clear” mentioned by the reviewer. We think that these cases retrieve interesting information not seen with just the abundance.

Additionally, there are many different measures of diversity that are commonly used for sequences (e.g. alpha diversity estimates often used in microbial ecology for 16S or functional genes), and I need a much more detailed reasoning why a metric that includes both abundance and diversity (some sort of product of these different measures) is more helpful to understand the community.

There are three different dimensions for each gene in our data: its abundance, its diversity (number of unique sequences) and the taxonomic assignment of these sequences. We have already considered abundance and taxonomy in Figure 2. This tells us which are the most abundant genes and, therefore, those likely to carry out a more important function in the environment. Thus, for example, the *amt* gene seems to be the most widespread for acquisition of nitrogen. It also tells us which taxa are the more abundant. In the example of the *amt* gene in the epipelagic, it is obvious that alpha, gamma proteobacteria and cyanobacteria are the main contributors. However, in the mesopelagic the main contributor is *Nitrososphaeria*.

This is certainly informative and interesting. However, by considering diversity of sequences we go a step further. The plots in Figures 4 and 5 have the virtue of combining the three dimensions in a visually clear fashion (we hope). We realize the first confrontation with the plots may be confusing. But once familiarized with them, it is extremely easy to compare different environments and detect generalities and departures from them in any of the three dimensions (synthesizing a lot of information). The reasoning behind these calculations is that diversity of sequences of a gene contains interesting information not seen in the abundance data. This is actually one of the main points of our work.

If α-diversity were to be computed at sample level, would be larger where there are more taxa, but it would not say whether gene is more or less diversified than expected at its abundance (by taxon by context). Our parameter δ captures that deviation; k then weighs it by abundance. If calculated in taxonomic levels, would be synonym with d_obs.

We improved the explanation of the parameters overall.

I recommend the authors re-structure their manuscript, starting with the abundance (and what can be learned from it), and only then introducing the importance of diversity, and where it contributes another layer to the understanding of the guilds.

We took the reviewer advice and rewrote some paragraphs clarifying this out, mainly at the end of 2.2, and the new 2.3 (old 2.4), and we restructured the manuscript, introducing abundance results, diversity results, and then results with both measures. We hope these concepts now flow better with the narrative.

3) The authors focus on set of genes (lines 420-422), but expect the reader to know what pathways these genes belong to. When the results are shown (e.g. in Figure 2), these genes are ordered alphabetically and not, for example, by function. I think a good first step would be to present these genes in an organized manner, perhaps as figure 1, explaining which pathway each gene relates to. Then, in subsequent figures, the genes could be organized in such a way as to make analysis of the functions more clear (as the authors did quite nicely in figures 3 and 4).

We did not think it appropriate to add a figure with all the biochemical pathways, since they are well known. We did provide the names of the pathways for each gene in Table S1. As the reviewer mentions, we could the pathways as an outer ring in the polar plots. The genes in Figure 2 are in alphabetical order for easy location.

4) The summary figure is unclear to me, showing mostly functions that change in the water column between two individual stations and the mean of the others. I think these are things that have been studied intensively (certainly in terms of N cycling in OMZs), and I don’t think there is a detailed-enough comparison of the authors’ findings in this regard and previous literature.

We agree with the reviewer. We tried to summarize a lot of information, but the figure needs a better explanation to be understood. We added a new section (new 2.5) covering this, and to actually refer to the remarks displayed in figure 6 (which are qualitative comparisons of the old figures 3 and 4).

If the focus here is on guilds, perhaps a figure should be generated specifically asking which organisms do what, or which organisms are part of which “guild” (i.e. share genes) – and again, comparing these results with the literature.

This is precisely what figures 3 and 4 do. Does the reviewer want a table with all the taxa contributing to each gene?
These are the main contributors to the main N-related guilds, listed by K-contribution. Figure 6 is a summary of these, focused on the differences between 120 and 141 (N:P ratios).

Some additional specific remarks:

Abstract (line 23) and elsewhere – I don’t think there is any added gain by shortening “Malaspina” to “MP” (acronyms often only add confusion).

Done as requested.

Lines 64-70 seem more relevant to the materials and methods.

We moved part of these lines to methods, specifically: “Sampling was done with Conductivity-Temperature-Depth (CTD) profilers with Niskin bottles from the surface to 4,000 meters, analyzing them for physical, chemical, and biological parameters.” The other parts related to depth stratification were left for (what we believe) essential context.

Line 99, Figure 1: What do the authors mean by “N:P”? Is this NOx:PO4? NO3:PO4? TDN:TDP? Also, were all of the measurements used to calculate these ratios above the limit of detection of the instrument (especially at the surface)?

We now clarify that we use NOx:PO4 ratios.

The reviewer points out a potential problem, since NOx levels at the epipelagic are usually very low. If a value is indicated, however, it means that it was above the limit of resolution of the technique. Some of the measurements in Malaspina were indeed under the detection limit, however, every sample sequenced and used in this study has a NOx:PO4 value attached.

I would also plot Figure 1B as a classical oceanographic depth profile (so depth on the Y axis, N:P ratio on the X).

We appreciate the suggestion. We kept the horizontal depth axis for three reasons. The first two are aesthetic. First, it preserves visual consistency with figs 4 and 5, where depth strata are read left-to-right; this makes it easier to cross-reference Figure 1B with subsequent panels that stratify by depth. Second, the horizontal layout improves readability when plotting all 11 stations together. This avoids vertical compression of the upper 500 m (as it is B panel where panel A is horizontal). Third, the true predictor variable is Depth, not N:P (which is treated as a response variable to depth).

Nevertheless, we showed the classical oceanographic depth profile in schematic fig. 6, and now additionally in the novel supplementary fig S1

Lines 98-101 – O2 could also affect the results (a “confounding factor”), but I don’t think the authors discuss this option much (perhaps I missed it?).

We think the reviewer has a point: O2 is indeed a major factor to consider. However, temperature, salinity, conductivity, and many other variables can be also confounding factors. From these, we’ve only interrogated depth and N:P. We asked whether they influence the microbial functional repertoire. But this selection is not arbitrary: (i) depth is chosen because it captures covariance of the other important factors (e.g. pressure, salinity, temperature, oxygen, DOM, etc., as shown in new fig S1), (ii) N:P is chosen because we are investigating N-related guilds.

We think the reviewer’s argument merited a clarification in the text, which is now available at the end of the first results section.

Lines 105-108 – how do these measurements of the N:P ratio compare with other studies of this ratio across the oceans? Could these be related, for example, to global deep water circulation patterns?

We have compared our N:P ratios to Redfield ratio which shows whether the systems is more limited by N or by P. The values of this ratio change around those of the Redfield ratio in the whole ocean.

Malaspina sampling was not designed to analyze water circulation patterns and, moreover, we only have a selection of all the stations. Therefore, we do not think we can say anything significant about circulation

Lines 122-127 – I like that the authors are being careful with their data, and want to focus on “bona fide” sequences, but can they show that when lots of sequences are being removed (e.g. the hzsA-III clade) these sequences are not orthologs from a previously unknown group, rather than paralogs or just genes with similar sequences (e.g. due to common domains)?

This is a problem that cannot be solved. There will always be (at least during our lifetimes) microbes that have not been described and therefore with unknown genes. The so called “dark microbial matter”. The only approach to solution is obtaining more sequences, more pure cultures, and carrying out the appropriate genetic and metabolic studies.

Moreover, orthology delineation is an evolving issue (for which each software uses slightly different criteria). So, even with the same data set, future bioinformatics tools will be able to extract more information. We have to work with what we have. And we have done our best collecting every metagenomic query placed in the trees (and every reference tree used, alongside with the used references and criteria for tree annotation). This information is freely available at zenodo as part of the supplementary materials for this work.

Lines 150-155 – this is a nice interim summary. Has this been shown in the past (e.g. very high abundance of ammonium transporters)?

We are not aware of prior studies that have documented these specific patterns. We added the latter statement. We also added a reference contextualizing our findings.

Line 380 – some citations seem to have a different format

It was probably due to a poor latex conversion. Should be solved now.

Line 392 – Climate (seasonal) variation may have a smaller impact than in other regions but is it really “minimal” (e.g. in relation to N:P ratios)? I think some citations of seasonal studies in similar latitudes are needed to make this statement more robust.

We modified the text a bit, since it was not our intention to diminish seasonal variation saying “minimal”. We now state: “…climate variations are expected to have a limited impact…”

Table 1 (and throughout the manuscript) – I’m not a statistical expert but I did not see many places where differences in abundance or diversity that were mentioned were associated with a statistical test. I realize that the authors have two specific depth profiles which they compare with “all the rest” but I think some sort of test should be made. Table 1 (the results of the GLM) seems to be limited to an overall comparison of the variation rather than specific genes or functions.

The GLM is indeed an overall comparison. In old fig S5 we have a krustal-wallis test comparing more specific contexts for N redox and N uptake genes. However, these tests are also very broad in the context of specific genes. Regarding specific genes, k-values and deltas are composite summary statistics that integrate abundance and deviation-from-expected richness. Even when only considering the abundance, as each context in our data is composed of several samples, Features Per Million of each marker gene are expressed as averages (Table S2).

Nevertheless, we now add a new supporting figure S7 with a new statistical test (one-sample t-tests of the mean against delta 0), showing whether epipelagic, mesopelagic or bathypelagic (without considering specific columns) do account for an increased diversity of each of the marker genes under study (showing z-scores).

Figures 2 -4 – what are the letter followed by an underscore (e.g. “c_” or “d_”) at the beginning of the taxa names?

They stand for the taxonomic level of the tag; d is for domain, c for class, etc. These are direct outputs from the python scripts.

How are the taxa organized in the two figures (what order)?

The order is by total abundance, and it is an automatic order that the plotting script assumes.

In figures 3 and 4, could they be organized by, for example, the “guilds”, and then colored in a way that can help highlight this?

They could be arranged in the reviewer’s way. However, as mentioned, we are summarizing three dimensions: taxonomy, abundance and diversity. We think the ordering the by abundance helps identifying the taxonomy of the sectors in the radial plots

Also, some of the taxa in Figure 3 are unclear (e.g. “Casp-alpha…”). What is the taxonomic rank (or ranks) shown?

These are automatic taxonomic labels made by the metagenomic pipeline used. The sequences are compared with databases and the software tried to provide the most detailed taxonomy possible. With metagenomes, the taxonomic level is not very specific.

Also in Figure 3 – where is Urea found (it is not in any of the groups – non-AA organic N, AA organic N and inorganic N).

This is because ureC, together with amt, ilvC and nirK were too abundant and diverse to be compared in the radial plots, even in a log scale. If represented the remaining genes would be hardly visible. We now explicitly state that we do not analyze the diversity of every N-related gene.

Figure 5 – the black arrows show change compared to what?

We assume that the reviewer is referring to figure 6. The black arrows show a functional departure of this layer in the stations 120 or 141 with respect to the average ocean. However, they are not “blue” or “red” because this feature is found at least in other water column, so these properties are not unique to this station. It is a way to be conservative. For example, in the bathypelagic of st. 120, we find a depletion of nitrification functions compared to the ocean average. However, this is not a “unique feature” of the st. 120 bathypelagic. This is now better clarified in the figure legend.
